# A multi-stakeholder survey on communicating cost-effectiveness uncertainties to stakeholders: a case study of ICER

**DOI:** 10.1093/haschl/qxag009

**Published:** 2026-01-19

**Authors:** Jan-Willem Versteeg, Wim G Goettsch, Aukje K Mantel-Teeuwisse, Daniel Ollendorf, Christine Leopold

**Affiliations:** Division of Pharmacoepidemiology and Clinical Pharmacology, Utrecht Institute for Pharmaceutical Science, Utrecht University, Utrecht 3584 CG, The Netherlands; Division of Pharmacoepidemiology and Clinical Pharmacology, Utrecht Institute for Pharmaceutical Science, Utrecht University, Utrecht 3584 CG, The Netherlands; Zorginstituut Nederland, Diemen 1112 ZA, The Netherlands; Division of Pharmacoepidemiology and Clinical Pharmacology, Utrecht Institute for Pharmaceutical Science, Utrecht University, Utrecht 3584 CG, The Netherlands; Center for Evaluation of Value and Risk in Health, Tufts Medical Center, Boston MA 02111, United States; Division of Pharmacoepidemiology and Clinical Pharmacology, Utrecht Institute for Pharmaceutical Science, Utrecht University, Utrecht 3584 CG, The Netherlands

**Keywords:** cost-effectiveness analysis, health technology assessment, uncertainty, uncertainty communication

## Abstract

**Introduction:**

Cost-effectiveness analyses (CEA) are integral to health technology assessments performed by the US-based Institute for Clinical and Economic Review (ICER). This study aims to examine how stakeholder groups perceive and interpret ICER's communication on uncertainty in CEA findings.

**Methods:**

We performed a multi-stakeholder survey among individuals recruited via ICER's weekly update email (March/April 2025). The survey focused on 6 topics related to ICER's uncertainty communication based on the organization's published reference case, consisting of Likert-scale and open-ended questions. Results were analyzed descriptively with thematic analysis of open-ended responses.

**Results:**

Thirty-four responses were collected, representing different stakeholder groups. Results indicated that 16/18 of respondents found sensitivity analyses to be communicated clearly (varying by analysis type), while 9/17 found structural and parametric assumptions to be communicated clearly. Most report sections were understandable respondents, and 15/23 found the communication of uncertainty outside reports to be understandable. Thematic analysis identified scenario analyses, structural/parametric uncertainty communication, and stakeholder-tailored communications as areas for future enhancement.

**Conclusions:**

While methodological guidelines focus on quantifying uncertainty, limited guidance exists for communicating key facts on uncertainty to different stakeholder groups. This study highlights specific stakeholder-perceived areas for improvement. Future research could compare these results to other organizations.

Key points

- While scientific literature and methodological guidelines extensively cover how to quantify uncertainty in cost-effectiveness analyses, only limited information exists on how to communicate that uncertainty to different stakeholder groups. This study examined how ICER's stakeholders perceived uncertainty communication from cost-effectiveness analyses.
- ICER's uncertainty communication was generally well-understood by its stakeholders, with 65%-82% (between 11/17 and 14/17) of stakeholders finding most report sections understandable and 80% (16/18) finding sensitivity analyses clearly communicated. However, the mixed results across different areas, particularly lower levels of understanding of the clarity of communication on the impact of structural and parametric assumptions, indicated that further enhancement is possible.
- Stakeholders consistently identified 3 specific improvement opportunities in their responses to open-ended questions: enhancing scenario analyses, strengthening communication around structural and parametric uncertainties, and developing stakeholder-tailored communications.

## Introduction

Healthcare systems aim to provide access to innovative medical treatments while managing increasing healthcare expenditures that are putting strain on budgets.^1,2^ Because of this, policymakers must decide which interventions to reimburse, while balancing investment with the potential to improve population health. To make these complex tradeoffs, health technology assessment (HTA) is used to systematically evaluate the value of new therapies compared with the current standard of care.^3^ Relative effectiveness assessment (REA) is often the starting point of an HTA. It determines the extent to which an intervention does more good than harm, compared with one or more alternative interventions under the usual circumstances of health care practice. Cost-effectiveness analyses (CEA) are often performed in addition to REA to assess whether the clinical benefit is in balance with the costs of the new therapy.^4^

CEAs are typically based on health economic simulation models to make this comparison, providing quantitative evidence beyond the results of clinical studies. However, these models are often built upon limited and imperfect data from multiple sources, creating uncertainty that can significantly impact policy decisions.^5^ Uncertainty in CEAs originates from parameter uncertainty (eg, imprecision in input data from clinical trials, real-world data, or expert opinion), structural uncertainty (eg, model design choices and the health states used), and methodological uncertainty (eg, the discount rate used, the time horizon used, and the modeling perspective)^6-8^. This uncertainty introduces financial risk for health systems: if reimbursement decisions are based on imprecise cost-effectiveness estimates, payers may commit to high prices for technologies whose actual value could be substantially lower than modeled. At the same time, the uncertainty complicates reimbursement decision-making, hindering timely patient access to potentially life-saving treatments. If not explicitly addressed and properly communicated, uncertainty in CEAs imposes financial and clinical risks to health systems when informing coverage, access, and reimbursement decisions.

In the United States (US), the Institute for Clinical and Economic Review (ICER), a non-profit, independent research organization, conducts HTAs to inform both private and government payers.^9^ In addition to their REA, their reports include both CEA and budget impact analyses. Since ICER aims to publish its HTA assessment as close as possible to the granting of marketing authorization by the US Food and Drug Administration (FDA), its assessment is often the earliest HTA available for a new medicine.^10^ This means ICER assessments can be valuable tools for informed decision-making by US-based payers, but it also means their evaluations are often conducted at a time in the medicine lifecycle when there is only limited available data. Therefore, it is crucial that ICER clearly and transparently communicates the uncertainty in its CEA to its stakeholders to support well-informed coverage and reimbursement decisions. As a widely recognized organization in HTA transparency and methodological rigor, ICER provides an informative case for examining the challenges of communicating uncertainty. Learnings from this case study will be relevant across other HTA organizations.

How to quantify uncertainty in CEAs is described extensively in scientific literature. Studies describe in detail what types of sensitivity analyses could and should be performed to deal with parametric, structural, and methodological uncertainty.^6-8,11^ In addition, HTA organizations often have specific sections added to their guidelines and/or reference cases about quantifying uncertainty in CEAs. They describe step by step what sensitivity analyses need to be performed and how they should be displayed in HTA reports.^12-14^

Despite extensive guidance on quantifying uncertainty in CEAs, guidelines and scientific literature offer limited direction on communicating this uncertainty, including which communication formats should address it and the appropriate level of detail. ICER communicates to its stakeholders mainly through written communications such as its main evidence report, the “report at a glance” (a policymaker-oriented summary), the “patient snapshot” (a plain-language summary with specific information for patients), and policy papers. This study aims to examine how ICER stakeholders perceive and interpret this information. To inform recommendations for increasing the clarity and understandability of uncertainty communication in HTA practice, we conducted a multi-stakeholder survey examining ICER's written communications as a case study, with implications for uncertainty communication across HTA organizations.

## Data and methods

### Research method

We conducted a structured online survey among ICER stakeholders. The survey was open for 6 weeks, from March 14th to April 25th, 2025. The survey was open to all respondents, both US-based and international, and focused on 6 topics related to ICER communication on uncertainty, specifically uncertainty in their CEA or clinical uncertainty impacting their CEA.

### Participant selection

Participants were recruited through ICER's stakeholder mailing lists, while researchers did not have access to the stakeholders' email addresses to protect privacy. The survey invitation was included twice in ICER's weekly newsletter (*N* = 8834 subscribers), sent out on March 14 and March 28, 2025. The survey was also shared via the researchers' LinkedIn profiles to broaden outreach. Stakeholder groups of interest included: private payers, health technology developers, healthcare providers, healthcare consultants, HTA organizations, patient organizations, governmental payers (eg, Veterans Affairs, Medicare, and Medicaid), and academia. Both US-based and international stakeholders were invited to fill out the survey. Survey responses were collected anonymously, and no incentives or compensation were provided to participants. Incomplete surveys were excluded from the analyses. All respondents had to agree to a consent form (Appendix 1) before starting the survey. In addition to the consent form, an information sheet on uncertainty in cost-effectiveness analysis and sensitivity analyses was provided to respondents as background information (Appendix 2).

### Survey construction

Our survey consisted of 6 key topics. Background information of respondents was limited to their occupational background and familiarity with CEAs and ICER reports. Topics were selected based on ICER CEA processes, its reference case,^14^ and reporting structure, aiming to capture all of ICER's current communications of uncertainty in their CEA. Key topics were (1) background information, (2) familiarity with CEA and written ICER communications (evidence report, report a glance, patient snapshot, policy papers, and email communications), (3) CEA base-case results and sensitivity analyses, (4) structural and parametrical uncertainty, (5) understanding different sections of an ICER report, and (6) understanding ICER communication outside of reports. Topics 1 and 2 used multiple-choice questions, while topics 3 through 6 used 5-point Likert scale items followed by open-ended questions. A 5-point Likert scale was selected based on evidence that 5-point scales demonstrate adequate reliability while being easy for respondents to complete quickly.^15^ The 5 response options were: strongly agree, somewhat agree, neither agree nor disagree, somewhat disagree, and strongly disagree. This odd-numbered scale offers a neutral midpoint option, enabling respondents to express genuine ambivalence rather than being forced to make a directional response. A “not applicable” option was included in all multiple-choice questions to improve response validity. Respondents who indicated that they did not read the full ICER reports were redirected to the survey question about communication outside of the reports. In addition, only parts of questions 1 and 2 were mandatory, allowing respondents to skip questions that they felt did not apply to their experience or expertise. The survey was created and conducted using Qualtrics® (Qualtrics, Provo, UT, USA) survey software. The survey was first pilot-tested internally at ICER and updated based on comments from this pilot test before being sent out to respondents. The full survey and survey logic can be found in Appendix 3.

### Data analysis

Survey data were analyzed using descriptive statistics for quantitative items and thematic analysis for open-ended responses. Given the exploratory nature of the study and the unknown response rate (because of the way the survey was shared with participants), no inferential statistical tests were conducted. Descriptive statistics were performed using the Qualtrics software and visualized using Microsoft Excel. Thematic analysis of answers to open-ended questions was performed using Microsoft Excel. In the thematic analysis, results from the open-ended questions were coded into recurrent theme categories by author JV. Following this, the most frequent recurring themes were identified, and coded survey responses were further examined to explain the themes in this article.

### Ethical approval

This study received ethical approval from the Tufts Medical Center Institutional Review Board (IRB; Study ID: STUDY00005770).

## Results

In total, 34 completed responses were recorded in the survey window (Table 1). Respondents were from a wide range of stakeholder groups, with most respondents working in the consultancy or academic sectors. No respondents were employed by private payer organizations; however, some were affiliated with government agencies involved in reimbursement and payment processes, such as the Department of Veterans Affairs or Medicaid. Most respondents indicated that they were either very familiar (33%) or extremely familiar (57%) with CEA. A total of 60% of all respondents routinely read ICER evidence reports, followed by ICER policy papers, emails, and “reports at a glance” (all 40%-50%). Of the people reading ICER reports, 56% read the full report, with lower percentages reading specific subsections of the report. As respondents were able to skip questions that they felt did not apply to their experience, questions received responses from subsets of our full respondent population. The sample of 34 respondents, recruited through ICER's newsletters, demonstrates high familiarity with CEA and routine engagement with ICER materials, likely representing more sophisticated users than the broader stakeholder population. Despite a limited sample size, if these methodologically-informed stakeholders identify communication challenges, audiences with less technical knowledge would likely face greater difficulties.

**Table 1.** Respondent characteristics of survey respondents are displayed in numbers (*N*) and percentages.

Respondent characteristics	*N*	(%)
Total number of completed responses	34	
**Stakeholder groups**		
Healthcare developer	4	13%
Health care provider (eg, hospital, outpatient clinic)	1	3%
Healthcare consultancy	6	20%
Health Technology Assessment organization	4	13%
Patient organization	1	3%
Academia	8	27%
Governmental agency (eg, Veterans Affairs or Medicaid)	3	10%
Other	3	10%
**CEA familiarity**		
Slightly familiar	2	7%
Moderately familiar	1	3%
Very familiar	10	33%
Extremely familiar	17	57%
ICER communications read*		
ICER Evidence Reports (ie, Draft, Revised, or Final)	18	60%
ICER policy papers	16	53%
ICER emails	14	47%
ICER patient snapshot	2	7%
ICER Report at a Glance	13	43%
None	2	7%
**ICER reports sections read** ^a^		
The full report	10	56%
The executive summary	9	50%
The comparative clinical effectiveness section	7	39%
The long-term cost-effectiveness section	5	28%
The benefits beyond health and special ethical priorities	4	22%
The health benefits price benchmark	4	22%
The potential budget impact	5	28%
The policy recommendations	8	44%
Only very small targeted sections	2	11%
**Frequency of ICER communication use**		
Weekly	2	7%
Monthly	8	29%
Less than monthly	7	25%
Once or twice a year	9	32%
Not at all	2	7%

Important to note is that participant had the opportunity to skip most questions, and as such, not all questions were answered by all participants. CEA, Cost-effectiveness Analysis; ICER, Institute for Clinical and Economic Review. aRespondents were able to answer multiple questions (*N* = 34).

**Figure 1.**
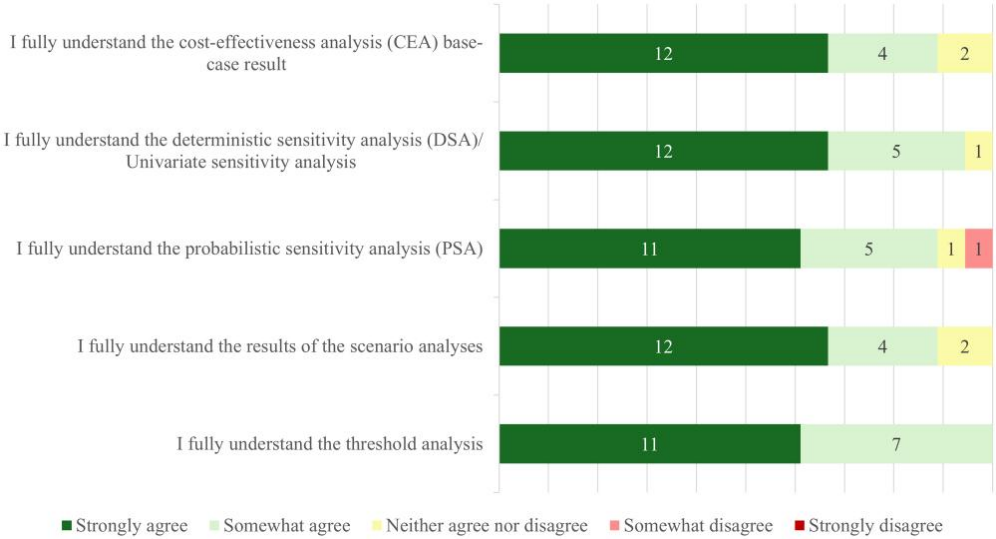
Understandability of sensitivity, scenario, and threshold analyses as presented by ICER in their reports (*N* = 18).

**Figure 2.**
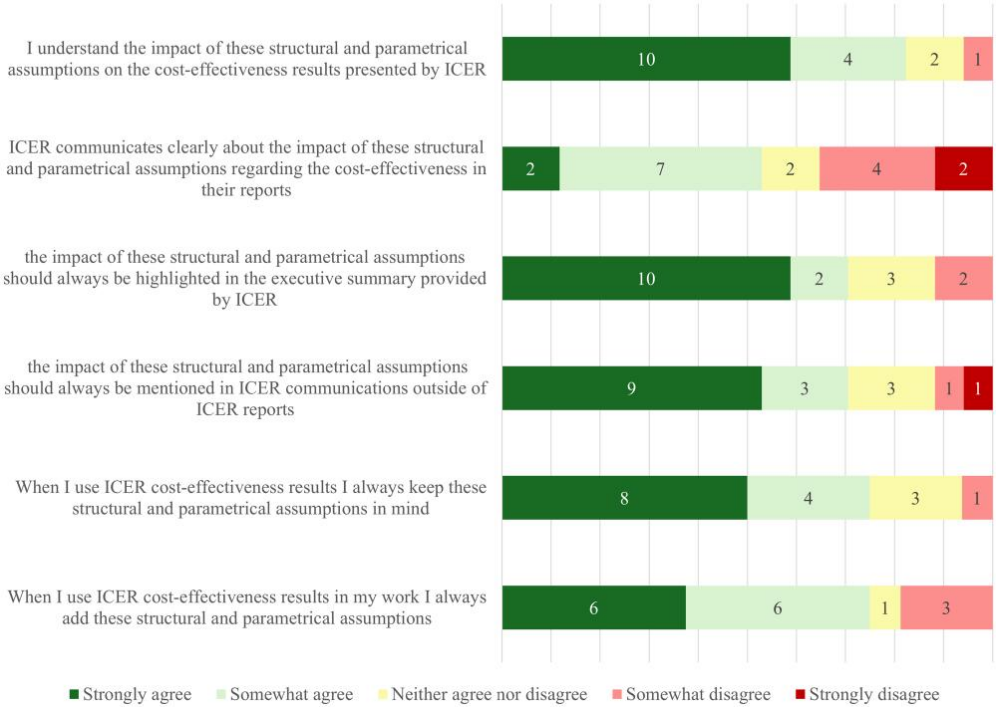
Understandability of structural and parametric assumptions and their impact on the results as presented by ICER in their reports (*N* = 17).

**Figure 3.**
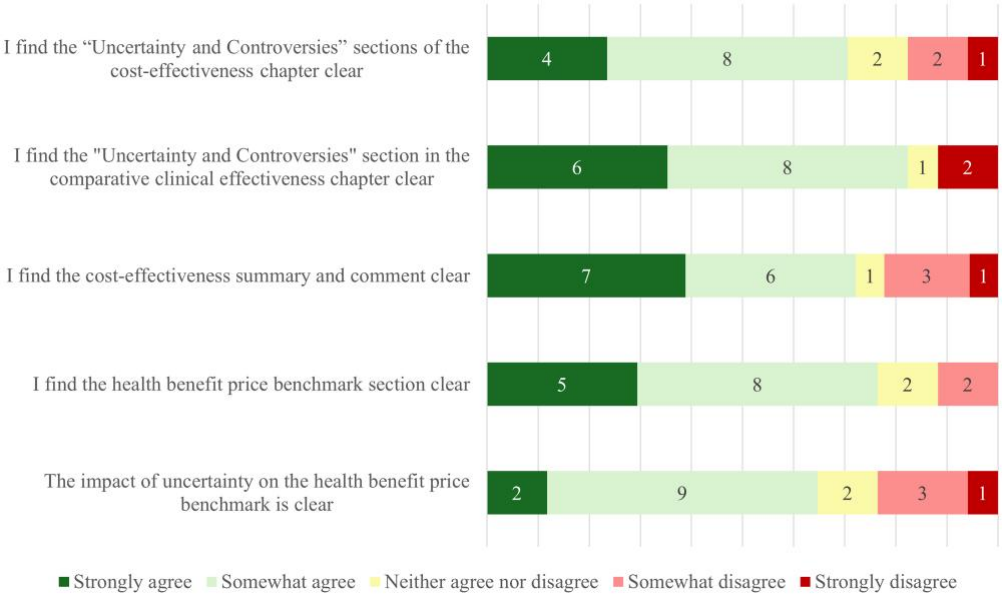
Understandability of different ICER report sections (*N* = 18).

Responses to the Likert-scale questions indicated that overall, respondents agreed that ICER communications were understandable and clear, as depicted in Figures 1-3. Targeted questions regarding the performed sensitivity, scenario, and threshold analyses are presented in Figure 1. These results showed that most respondents at least somewhat agreed (>85% (16/18), of which >60% (11/18) strongly agreed) that the sensitivity, scenario, and threshold analyses were understandable.

Questions targeting the understandability of structural and parametric assumptions and their impact on the CEA are presented in Figure 2. Respondents did, for the most part, understand the presented structural and parametric assumptions. However, some respondents at least somewhat disagreed (24% somewhat (4/17), 12% strongly (2/17)) that the communication about the impact of these assumptions was clear. Approximately 70% (12/17) of respondents indicated that they kept the structural and parametric uncertainties in mind when using ICER's work. Around the same number of respondents said that they would always add the structural and parametric assumptions in their work when they incorporated ICER results. When asked about different sections of the ICER reports, between 65% (11/17) and 82% (14/17) of respondents at least somewhat agreed that all sections surveyed were understandable, with between 10% and 40% strongly agreeing depending on the report section (Figure 3). Respondents were most often critical about the impact of uncertainty on the “price benchmarking” section and on how clear the “summary and comment” section of the report was, with approximately 20% (4/17) stating that they did (partially) not agree that the sections were clear. In addition to the results in Figures 1-3, respondents were asked about ICER communications outside of ICER evidence reports. The responses to this question showed that 65% (15/23) of respondents agreed at least partially, of which 35% strongly (8/23), that the communications about uncertainty in the CEA in other communication methods, such as emails and “reports at a glance”, were presented clearly.

In response to the open-ended questions, there were some recurrent themes that were mentioned throughout the survey. The most often-recurring responses were coded into 3 overarching themes with several subthemes, as described below.

### Enhanced scenario, sensitivity, and threshold analyses

First, an often-recurring theme was the call for more robust scenario analyses to better highlight and communicate the uncertainty. In addition, greater justification for scenario analyses could be provided and could be more stakeholder-informed.

*“**More robust scenario analysis should be conducted based on stakeholder engagement;** more focus should be placed on the one way sensitivity analysis (OWSA) in the model presentation to better characterize drivers of model results; too much emphasis is on the threshold analysis and **not enough emphasis is on using the model as an important tool to pressure test assumptions/conclusions** and to guide broader discussion (ie, voting and policy roundtable) and the overall report conclusion.”*

Second, respondents mentioned themes related to improved transparency and interpretability, for instance, in the form of standardized visualizations of the outcomes of the sensitivity analyses.

Third, respondents called for less emphasis on point estimates (ie, “a number and a price”) and threshold analysis, and suggested focusing on using the model to pressure test assumptions and to highlight uncertainties.

*“Less emphasis on base case and point estimates, and increase in emphasis in highlighting areas of uncertainty, perhaps leading with the areas of uncertainty rather than the base case and value-based price. A lot more scenario analysis is needed and highlight how uncertainty can be reduced outside of just varying price. Consistently utilize visualization of uncertainty analysis, eg, tornado diagrams, CEAC curves, etc. Utilize the full richness of uncertainty, not just one or a few variables, and utilize more than the threshold analysis alone.”*

### Communication of structural and parametric assumptions

First, multiple respondents would welcome more discussion on the impact of these assumptions on the results of the CEA in ICER reports. Respondents asked for robust and transparent documentation on the justification of these assumptions, but even more so on what their impact could be on the presented results. A respondent called this critical as it would ensure that stakeholders can interpret the results accurately and can use the results to make informed decisions.

Second, in line with this reasoning, there was a recurrent call to add these structural and parametric assumptions to the executive summary, as this is sometimes the only section that many stakeholders read. One respondent also called for the uptake of these assumptions into external communications, but, contrastingly, multiple other stakeholders mentioned that, although the information is critical, it might not be understandable and beneficial for all stakeholders.

*“Reports and presentations need more robust and transparent documentation on how these assumptions could impact the results. This could include insights on how assumptions may be biasing or limiting the model's ability to demonstrate product value.”*

### Increased communication of uncertainties outside of evidence reports and tailored communications to specific audiences

First, in the open-ended questions on specific ICER report sections and ICER communications outside of reports (eg, emails and reports at a glance), there was a broad interest in more information on uncertainty in ICER communications outside of ICER evidence reports. Respondents often mentioned that uncertainty should always be highlighted where results are presented.

Second, a theme only highlighted by one respondent, but important to mention, was the tailoring of communications to different stakeholder groups. It is in line with many other responses that called for more or less information in specific communication measures about specific uncertainties and assumptions.

*“Different stakeholders have varying levels of expertise and priorities. For example: Policymakers: Focus on high-level takeaways, decision thresholds, and budget implications. Clinicians: Emphasize clinical relevance, trade-offs, and how uncertainty might affect patient care. Patients/Public: Use relatable examples, avoid jargon, and focus on how findings impact health outcomes. Use Role-Specific Messaging: Develop separate materials for different audiences rather than relying on a one-size-fits-all approach.”*

## Discussion

This multi-stakeholder survey reflects the perspectives of 34 diverse ICER stakeholders on how ICER communicates uncertainty in its written materials. Overall, a majority of stakeholders found ICER's communications to be clear and understandable. However, stakeholders identified areas for improvement for specific report sections: the communication of how structural and parametric assumptions affect cost-effectiveness outcomes, the explanation of how uncertainty impacts price benchmarking recommendations, and the clarity of the “summary and comment” section.

Thematic analysis of open-ended responses provided deeper insights into stakeholder concerns. Recurrent themes included a desire for more robust scenario analyses (with less emphasis on threshold analyses and greater emphasis on model stress testing), clearer communication and justification of structural and parametric assumptions, and more tailored communication of uncertainty in specific sections and materials.

These findings reveal that stakeholder concerns are concentrated not primarily on understanding the sensitivity analysis, as most state that ICER communicates about these in an understandable and clear manner, but in grasping how structural choices and assumptions impact the model outcomes and how this can be communicated more clearly. Stakeholders' call for more scenario analyses over threshold analyses indicates that they want to understand model robustness and assumption sensitivity, not only model conclusions. This suggests that effective uncertainty communication requires not just reporting analytical results, but making the reasoning behind structural decisions and their implications for conclusions transparent and understandable to different stakeholder groups. While our survey demonstrates that stakeholders value clarity of uncertainty communication, we recognize that clear communication is necessary but not sufficient for ensuring accurate uncertainty characterization. Economic evaluations may contain biases in input data or structural assumptions that are difficult to detect. Most literature on CEA quality has focused on transparency, a necessary foundation for stakeholders to evaluate these potential biases critically. Understanding how structural choices affect outcomes enables this critical evaluation, though transparent communication does not guarantee that the communicated uncertainties accurately represent all analytical limitations.

Clear communication to HTA stakeholders becomes increasingly important as the HTA landscape evolves. Almost all assessments have to deal with one or multiple forms of uncertainty,^16^ for instance, with innovative and complex therapies, such as gene therapies, reaching the market with limited evidence and introducing new challenges for HTA.^17^ The evidence base with which new therapies reach the market is also decreasing generally, with medicines coming to market with single-arm pivotal trials, uncertainty regarding their long-term relative effectiveness, and only surrogate endpoints available^18-20^ On the other hand, policymakers have more tools at their disposal to address these uncertainties in the form of adjusted pricing based on value of information analysis and managed entry agreements^21-23^. Using these tools effectively depends fundamentally on policymakers' understanding of the uncertainty that exists. Therefore, HTA organizations should strive to communicate uncertainty in CEAs in a complete, transparent, and understandable way.^24^

HTA organizations worldwide face similar challenges in communicating uncertainty to diverse stakeholders. Current guidance emphasizes the technical execution of uncertainty analyses but provides limited direction on making uncertainty information accessible and interpretable for decision-making. Methodological guidelines from HTA organizations, such as the National Institute for Health and Care Excellence (NICE, United Kingdom), the Pharmaceutical Benefits Advisory Committee (PBAC, Australia), and Canada's Drug Agency (CDA-AMC, Canada), specify in detail which sensitivity analyses to conduct, which parameters to vary, and how to format probabilistic results.^12,25,26^ The CDA-AMC's guideline is the only one with a specific section on “reporting,” but it stays on a surface level and only states high-level aspects: reports need to be transparent and detailed, a summary written in non-technical language should be included, and CEA results should be presented in graphical or visual form in addition to tabular presentation.^26^ The scientific literature on CEA reflects similar priorities. Most articles focus predominantly on the more technical aspects of which sensitivity analyses to perform, when, and how the results of these sensitivity analyses should be displayed^6,7,11,27-29^ An often-reported guideline for CEA is provided by the US 2nd Panel on Cost-Effectiveness in Health and Medicine.^30^ This reporting guideline is brief on the recommendations for reporting on uncertainty and only states, in line with HTA organizations' guidelines, that there should be a visual representation of the results of sensitivity analyses and that the results of sensitivity analyses should be discussed.^30^ What remains largely unaddressed in both the guidance of HTA organizations and scientific literature is how to communicate the decision-relevant implications of uncertainty, which assumptions matter most for conclusions, why particular modeling choices were made, and how to communicate uncertainty to match different stakeholder backgrounds.

A notable exception that provides a broader overview of the communication of uncertainty comes from a paper, based on the 2021 HTAi Global Policy Forum discussion, which presents important insights on how to communicate uncertainties beyond methodological guidance.^31^ Factors that are mentioned include separate presentation and disaggregation of clinical and economic data rather than only a combined cost-effectiveness ratio, that uncertainties should be presented visually, but also that summary representations of uncertainty (such as cost-effectiveness acceptability curves or expected loss curves) may be useful, and lastly, that results should be presented at an appropriate level, keeping health literacy in mind. The paper also mentions ways to improve the communication of uncertainty, such as learning from other industries and using innovations in communication science.^31^ In addition to the insights from the global policy forum, another source of information could be the more elaborate studies on uncertainty communication that have been and are being performed in the closely related medical regulatory science field^32-34^.

Whether the limited published guidance on uncertainty communication in CEAs completely reflects the actual gap in HTA practice remains unclear, as internal guidelines on communication might exist. However, stakeholder feedback from our survey reveals that even organizations widely recognized for methodological rigor and analytic transparency can still improve their communication on uncertainty. ICER is very transparent in publishing details on their cost-effectiveness analysis for HTA standards, and still, our survey was able to highlight specific areas where uncertainty communication could improve. This suggests that uncertainty communication represents an ongoing, field-wide challenge, not an organization-specific issue, but a fundamental challenge in HTA. This shows the value of our work as a case report that all HTA organizations can learn from.

The most important limitation of this study is the low number of respondents. Despite multiple messages and communication channels, a limited number of stakeholders participated fully. This hinders the external validity of our work. Due to the exploratory nature of our study, it did not prevent us from examining some important findings, but it did make it impossible to stratify results by stakeholder group. A second limitation of our work was the possibility of reporting bias. Stakeholders filled out a survey on how well they understood certain aspects of uncertainty communication in CEA, but could overestimate their own experience and technical capacity. A final limitation of our work is the self-selected nature of the ICER mailing list used in our respondent recruitment. Stakeholders actively have to sign up for the mailing list, which could influence the results of our survey, as it might not have reached all relevant ICER stakeholders.

This exploratory case study offers insights into uncertainty communication challenges that can extend beyond ICER. The consistency of stakeholder responses around specific issues suggests patterns worth considering. Among our respondents, 3 themes emerged that could inform HTA communication practice. First, stakeholders indicated that structural assumptions could be more prominently available in summarizing and concluding report sections and communication outside of reports, not only in the technical result sections. Second, there was a preference among stakeholders to perform more scenario analyses, stress testing the model, and less focus on predominantly threshold analysis. Third, because of different stakeholder backgrounds and expertise, communication about the uncertainty in CEAs should aim to be tailored to their different needs.

These findings suggest several research directions for future research. Multi-organizational comparative studies could assess whether the findings in our case study are generalizable to other organizations. Secondly, larger sample size surveys would open up the possibility to stratify on stakeholder groups and formulate stakeholder group-specific recommendations. Thirdly, in-depth interviews with relevant stakeholders could help in further explaining the results of our study and give more depth to our recommendations. Fourth, given the limited participation from certain stakeholder groups in our survey, particularly private payers, future research should prioritize their perspectives on uncertainty communication needs. Finally, while our study addresses how communicated uncertainties are understood, effective transparency also depends on which uncertainties are quantified for communication. Future research should investigate which uncertainties remain unquantified or inadequately captured by current sensitivity analysis practices, examining how organizational choices and methodological constraints shape these decisions about what to pressure test quantitatively vs describe qualitatively.

## Conclusion

This exploratory study of 34 ICER stakeholders found that respondents understood the presented sensitivity analyses, but wanted more emphasis on scenario analysis, impact of uncertainty, and stakeholder-tailored communication. While limited in scope, these findings reveal uncertainty communication challenges that are not adequately reflected in current HTA guidance or literature. As therapies with substantial evidentiary uncertainty become increasingly common, HTA requires communication strategies that translate analytical rigor into stakeholder comprehension. This work establishes a foundation for developing such approaches.

## Supplementary Material

qxag009_Supplementary_Data
