# Supplementary material for: A multi-stakeholder survey on communicating cost-effectiveness uncertainties to stakeholders: a case study of ICER
Source: Health Aff Sch. 2026 Jan 19;4(3):qxag009. doi: 10.1093/haschl/qxag009 (PMC12993711; doi:10.1093/haschl/qxag009)
Supplement: qxag009_Supplementary_Data [file qxag009_supplementary_data.zip › CEA_Uncertainty_Versteeg_Appendix 1-3.pdf]

Appendix 1:

Consent Information Sheet

Multi-Stakeholder Survey on Communicating Uncertainties in Health Technology Assessment

You are invited to take part in a research study being conducted by Dr. Daniel Ollendorf and Jan-Willem Versteeg from Tufts Medical Center. We are inviting you because you get information from the Institute for Clinical and Economic Review (ICER).

Background:

Uncertainty is important in all cost-effectiveness analyses. These analyses look at costs and effects over a long time and use a lot of information. Some of this information is not certain and different methods or assumptions can also make the results less certain. In its reports, ICER shows this using sensitivity analyses. ICER tries to write about the most important uncertainties in a clear and useful way. **(If you want to learn more about this topic, check out our definitions document linked on the survey.)**

Purpose:

This survey will help us learn what you think about how ICER writes about the uncertainty in cost-effectiveness analyses. We will use your answers for research and to improve how we write about it.

Practical Information:

- The survey should take about 10 minutes.
- The survey has mostly multiple-choice questions. There are also a few open ones. Please answer them with a short response if you can, but if you have no suggestions, you can skip them.
- Your participation in this study is completely voluntary. You can choose to take part or not. You can also stop at any time without any penalty.

Data Protection:

There is a risk of loss of confidentiality, meaning your private information could be seen by someone outside of the research team. However, as we are not collecting identifying information about you, no one will be able to link your answers back to you. Please do not include your name or other information that could be used to identify you in your responses.

We will make every effort to keep your information private, but such privacy cannot be completely guaranteed, The Institutional Review Board of Tufts Medicine and Tufts University Health Sciences, may check records. This might include your research records. The records of this study might also be reviewed to make sure all rules and guidelines were followed.

We may publish the results of this research. However, we will not include any of your personal identifying information in the publication.

Your information that is collected as part of this research will not be used or distributed for future research studies, even if all of any identifiers you include are removed.

Other Information:

There are no direct benefits to you from taking part in this research. We cannot promise any benefits to others from your taking part in this research. However, results could help ICER to write more clearly about the uncertainty in the results of their analyses.

You will not be paid for your participation.

If you withdraw or are withdrawn from the study, any data collected from you before your withdrawal will not be used for the study.

Contact Details:

If you have questions, concerns, or complaints, or think the research has hurt you, please reach out to Jan-Willem Versteeg, MSc at Jan-willem.Versteeg@tuftsmedicalcenter.org or Daniel Ollendorf, PhD at Daniel.Ollendorf@tuftsmedicine.org.

If you have questions about your rights as a research study subject, call the Tufts Medical Center and Tufts University Health Sciences Institutional Review Board (IRB) at (617) 636 7512. This study has been reviewed by the Tufts Health Sciences IRB.

Appendix 2:

Definitions of terms used in the survey

Uncertainty:

When studying cost-effectiveness in healthcare, there is always some uncertainty. This is because we use different sources of data and models to predict costs and health outcomes over time. Understanding these uncertainties helps people make better decisions about healthcare. There are three types of uncertainty to know about.

Parametric uncertainty:

Parametric uncertainty happens when the data in a model is not exact. Information about costs and effects comes from different sources, like studies and price lists. Since this data is based on estimates, it is not always exact. This uncertainty can affect the results of a cost-effectiveness analysis.

Methodological uncertainty:

Methodological uncertainty happens when different ways of analyzing data could lead to different results. This could be due to the type of model used or the point of view taken in the evaluation. Using guidelines and best practices can help lower this uncertainty. ICER includes these guidelines in a "reference case," a set of standards on creating and estimating an economic model.[1].

Structural uncertainty:

Structural uncertainty happens when something does not clearly fit into other types of uncertainty. Bojke et al. describe it as uncertainty caused by one or more of the following[2,3]:

- Statistical models – How is data analyzed? For example, which risk factors are included?
- Evidence used – What data is included in the model? Are there missing pieces?
- Health conditions – Which diseases and stages should be considered?
- Treatment comparisons – What treatment options should be included?

Base-case result:

A base-case result is what we get when running a model using the most likely value for all input data and assumptions[2].

Deterministic sensitivity analysis (DSA):

A deterministic sensitivity analysis, or one-way sensitivity analysis, looks at how changing one input value at a time affects the results. It tests the highest and lowest possible values for each input value while keeping everything else the same. This helps show which uncertain input values have the biggest impact on the results[3].

Probabilistic sensitivity analysis (PSA):

A probabilistic sensitivity analysis (PSA) tests all uncertain input values at once by running many versions of the model with different values. These values are chosen randomly based

on a set range. The results show how confident we can be in the model's predictions, such as how often the PSA results in a cost-effective outcome[4].

Threshold analysis:

A threshold analysis finds the price where a treatment is cost-effective within a budget. ICER calculates prices that we would be willing to pay for effects. ICER uses limits of $50,000, $100,000, $150,000, and $200,000 per QALY (Quality-Adjusted Life Year) and evLYG (Equal Value of Life Years Gained)[1].

Scenario analysis:

This means testing different assumptions. Different values are chosen as input to see how they affect the results. For example, we might change costs, health benefits, or who is included to see how the results change in those situations.

Health benefit price benchmark:

All ICER reports include a health benefit price benchmark (HBPB). This analysis estimates acceptable treatment prices within a budget. ICER uses limits between $100,000 and $150,000 per QALY or evLYG gained for this analysis[5].

References:

1. Cost-Effectiveness, the QALY, and the evLYG. ICER. Accessed February 10, 2025. https://icer.org/our-approach/methods-process/cost-effectiveness-the-qaly-and-the-evlyg/

2. Base Case Analysis. YHEC - York Health Economics Consortium. Accessed February 10, 2025. https://yhec.co.uk/glossary/base-case-analysis/

3. Deterministic Sensitivity Analysis. YHEC - York Health Economics Consortium. Accessed January 28, 2025. http://yhec.co.uk/glossary/deterministic-sensitivity-analysis/

4. Probabilistic/Stochastic Sensitivity Analysis. YHEC - York Health Economics Consortium. Accessed February 10, 2025. https://yhec.co.uk/glossary/probabilistic-stochastic-sensitivity-analysis/

5. Value Assessment Framework. ICER. Accessed February 10, 2025. https://icer.org/our-approach/methods-process/value-assessment-framework/

Appendix 3:

Uncertainty communication ICER

Survey Flow

Standard: Survey information (1 Question)
Block: Topic 1: Background (3 Questions)
Standard: Topic 2: Familiarity Cost-effectiveness and ICER reports and communication (5 Questions)
Standard: Topic 3: cost-effectiveness analysis base-case result and sensitivity analyses (7 Questions)
Standard: Topic 4: Structural and parametric uncertainty (2 Questions)
Standard: Topic 5: Understanding different sections of the ICER report (6 Questions)
Standard: Topic 6: Understanding communication outside of ICER reports (2 Questions)

Page Break

Start of Block: Survey information

Q1.1 Dear Participant,   You are invited to take part in a research study being conducted by researchers at Tufts Medical Center. We are inviting you because you receive reports and other content from the Institute for Clinical and Economic Review (ICER).   In its reports, ICER illustrates uncertainty in cost-effectiveness analyses using quantitative techniques and text discussions. This survey will help us learn what you think about how ICER describes these uncertainties. We will use your answers for research on the best approaches for communicating uncertainty in these types of studies.   This survey will take about 10 minutes. There are both mandatory (marked with an asterisk) and optional questions. Participation in the survey is anonymous; none of the collected data will be linked to you. Please read our consent document before you start the survey: Survey consent document For more information on the topic of the survey please see our definitions document: Survey definitions document

○ I have seen the information sheet and understand the provided information (1)

End of Block: Survey information

Start of Block: Topic 1: Background

Q2.1 What kind of organization do you work for? Choose one of the following;

◯ Private payer (1)

◯ Healthcare developer (2)

◯ Health care provider (e.g. hospital, outpatient clinic) (3)

◯ Healthcare consultancy (4)

◯ Health Technology Assessment organization (5)

◯ Patient organization (6)

◯ Patient advocacy group (7)

◯ Academia (8)

◯ Governmental agency (e.g. Veterans Affairs or Medicaid) (9)

◯ Other: (10) ____

Q2.2 In a few words, what is your job/role in the organization where you work (e.g. patient, doctor, advisor, decision-maker, assessor)?

____

Q33 Are you currently based inside or outside the United States?

◯ I am based inside the United States (1)

◯ I am based outside the United States (2)

End of Block: Topic 1: Background

Start of Block: Topic 2: Familiarity Cost-effectiveness and ICER reports and communication

Q3.1 How familiar are you with the topic of cost-effectiveness analysis in general?

- Not familiar at all (1)
- Slightly familiar (2)
- Moderately familiar (3)
- Very familiar (4)
- Extremely familiar (5)

Q3.2 What research outputs from ICER do you read? Check **all** that apply.

- ICER Evidence Reports (i.e., Draft, Revised, or Final) (1)
- ICER policy papers (2)
- ICER emails (4)
- ICER patient snapshot (5)
- ICER report at a Glance (6)
- None (7)

Skip To: End of Survey If What research outputs from ICER do you read? Check all that apply. = None

---

Q3.3 How often do you use ICER research outputs in your daily work?

- ◯ Daily (1)
- ◯ Weekly (2)
- ◯ Monthly (3)
- ◯ Less than monthly (4)
- ◯ Once or twice a year (5)
- ◯ Not at all (6)

Display this question:

If What research outputs from ICER do you read? Check all that apply. = ICER Evidence Reports (i.e., Draft, Revised, or Final)

Q3.4 When using ICER evidence reports how much of the report do you read (multiple answers allowed)

- ▢ The full report (1)
- ▢ The executive summary (2)
- ▢ The comparative clinical effectiveness section (3)
- ▢ The long-term cost-effectiveness section (4)
- ▢ The benefits beyond health and special ethical priorities (5)
- ▢ The health benefits price benchmark (6)
- ▢ The potential budget impact (7)
- ▢ The policy recommendations (8)
- ▢ Only very small targeted sections (9)

Q3.5 What do you use ICER research outputs for in your work?

____

End of Block: Topic 2: Familiarity Cost-effectiveness and ICER reports and communication

---

Start of Block: Topic 3: cost-effectiveness analysis base-case result and sensitivity analyses

Display this question:

If What research outputs from ICER do you read? Check all that apply. = ICER Evidence Reports (i.e., Draft, Revised, or Final)

Q4.1 The statements below ask about your view of the base-case results and sensitivity analyses in the cost-effectiveness analysis. For more information on these analyses, please

see our definitions document: Survey definitions document   Please say how much you agree with the statements. If you do not look at one of the analyses, please select "Not applicable".

	Strongly disagree (1)	Somewhat disagree (2)	Neither agree nor disagree (3)	Somewhat agree (4)	Strongly agree (5)	Not applicable (6)
I fully understand the cost-effectiveness analysis (CEA) base-case result (1)	○	○	○	○	○	○
I fully understand the deterministic sensitivity analysis (DSA)/ Univariate sensitivity analysis (2)	○	○	○	○	○	○
I fully understand the probabilistic sensitivity analysis (PSA) (3)	○	○	○	○	○	○
I fully understand the results of the scenario analyses (4)	○	○	○	○	○	○
I fully understand the threshold analysis (5)	○	○	○	○	○	○

Display this question:

If The statements below ask about your view of the base-case results and sensitivity analyses in the... = I fully understand the cost-effectiveness analysis (CEA) base-case result [Neither agree nor disagree]

Or The statements below ask about your view of the base-case results and sensitivity analyses in the... = I fully understand the cost-effectiveness analysis (CEA) base-case result [Strongly disagree]

Or The statements below ask about your view of the base-case results and sensitivity analyses in the... = I fully understand the cost-effectiveness analysis (CEA) base-case result [Somewhat disagree]

Q4.2 What are ways to make the communication about the effect of uncertainty on the cost-effectiveness base case results clearer?

____

Display this question:

If The statements below ask about your view of the base-case results and sensitivity analyses in the... = I fully understand the deterministic sensitivity analysis (DSA)/ Univariate sensitivity analysis [Neither agree nor disagree]

Or The statements below ask about your view of the base-case results and sensitivity analyses in the... = I fully understand the deterministic sensitivity analysis (DSA)/ Univariate sensitivity analysis [Somewhat disagree]

Or The statements below ask about your view of the base-case results and sensitivity analyses in the... = I fully understand the deterministic sensitivity analysis (DSA)/ Univariate sensitivity analysis [Strongly disagree]

Q4.3 What are ways to make the deterministic sensitivity analysis/univariate sensitivity analysis clearer? in particular, how can the communication about the impact of uncertainty on these results be clearer?

____

Display this question:

If The statements below ask about your view of the base-case results and sensitivity analyses in the... = I fully understand the probabilistic sensitivity analysis (PSA) [Neither agree nor disagree]

Or The statements below ask about your view of the base-case results and sensitivity analyses in the... = I fully understand the probabilistic sensitivity analysis (PSA) [Somewhat disagree]

Or The statements below ask about your view of the base-case results and sensitivity analyses in the... = I fully understand the probabilistic sensitivity analysis (PSA) [Strongly disagree]

Q4.4 What are ways to make the probabilistic sensitivity analysis clearer? in particular, how can the communication about the impact of uncertainty on these results be clearer?

____

Display this question:

If The statements below ask about your view of the base-case results and sensitivity analyses in the... = I fully understand the results of the scenario analyses [Neither agree nor disagree]

Or The statements below ask about your view of the base-case results and sensitivity analyses in the... = I fully understand the results of the scenario analyses [Somewhat disagree]

Or The statements below ask about your view of the base-case results and sensitivity analyses in the... = I fully understand the results of the scenario analyses [Strongly disagree]

Q4.5 What are ways to make the scenario analyses clearer? in particular, how can the communication about the impact of uncertainty on these results be clearer?

____

Display this question:

If The statements below ask about your view of the base-case results and sensitivity analyses in the... = I fully understand the threshold analysis [Neither agree nor disagree]

Or The statements below ask about your view of the base-case results and sensitivity analyses in the... = I fully understand the threshold analysis [Somewhat disagree]

Or The statements below ask about your view of the base-case results and sensitivity analyses in the... = I fully understand the threshold analysis [Strongly disagree]

Q4.6 What are ways to make the threshold analysis clearer? in particular, how can the communication about the impact of uncertainty on these results be clearer?

____

Display this question:

If What research outputs from ICER do you read? Check all that apply. = ICER Evidence Reports (i.e., Draft, Revised, or Final)

Q4.7 In general, what are ways to make the outcomes of both the cost-effectiveness analysis, the sensitivity analysis, and the threshold analysis clearer? Please especially focus on the impact of model or data uncertainties on the results. (if answered before: skip this question)

____

End of Block: Topic 3: cost-effectiveness analysis base-case result and sensitivity analyses

Start of Block: Topic 4: Structural and parametric uncertainty

Display this question:

If What research outputs from ICER do you read? Check all that apply. = ICER Evidence Reports (i.e., Draft, Revised, or Final)

Q5.1 ICER reports include structural and parametric assumptions that affect the results. For example, a result might only apply if patients live at least five years or might depend on which other therapies were used to make the comparison. These structural and parametric assumptions appear throughout the document. For more background on types of uncertainty, see the definitions document: Survey definitions document Below are statements about

situations like this. They focus on structural and parametric uncertainty. Please share how much you agree with each one. ***This question is optional.***

	Strongly disagree (1)	Somewhat disagree (2)	Neither agree nor disagree (3)	Somewhat agree (4)	Strongly agree (5)	Not applicable (6)
I understand the impact of these structural and parametrical assumptions on the cost-effectiveness results presented by ICER (1)	○	○	○	○	○	○
ICER communicates clearly about the impact of these structural and parametrical assumptions regarding the cost-effectiveness in their reports (2)	○	○	○	○	○	○
the impact of these structural and parametrical assumptions should always be highlighted in the executive summary provided by ICER (3)	○	○	○	○	○	○

the impact of these structural and parametrical assumptions should always be mentioned in ICER communications outside of ICER reports (4)	o	o	o	o	o	o
When I use ICER cost-effectiveness results I always keep these structural and parametrical assumptions in mind (5)	o	o	o	o	o	o
When I use ICER cost-effectiveness results in my work I always add these structural and parametrical assumptions (6)	o	o	o	o	o	o

---

Display this question:

If What research outputs from ICER do you read? Check all that apply. = ICER Evidence Reports (i.e., Draft, Revised, or Final)

Q5.2 In general, how could the communication about the impact that these structural and parametrical assumptions have on the result of the analysis be improved?

____

End of Block: Topic 4: Structural and parametric uncertainty

Start of Block: Topic 5: Understanding different sections of the ICER report

Display this question:

If What research outputs from ICER do you read? Check all that apply. = ICER Evidence Reports (i.e., Draft, Revised, or Final)

Q6.1 The following statements are about other sections of an ICER report that talk about uncertainty. Please say how much you agree with each. If you do not read one of the options, please select "Not applicable".

	Strongly disagree (1)	Somewhat disagree (2)	Neither agree nor disagree (3)	Somewhat agree (4)	Strongly agree (5)	Not applicable (6)
I find the “Uncertainty and Controversies” sections of the cost-effectiveness chapter clear (1)	○	○	○	○	○	○
I find the "Uncertainty and Controversies" section in the comparative clinical effectiveness chapter clear (2)	○	○	○	○	○	○
I find the cost-effectiveness summary and comment clear (3)	○	○	○	○	○	○
I find the health benefit price benchmark section clear (5)	○	○	○	○	○	○
The impact of uncertainty on the health benefit price benchmark is clear (6)	○	○	○	○	○	○

Display this question:

If The following statements are about other sections of an ICER report that talk about uncertainty.... = I find the “Uncertainty and Controversies” sections of the cost-effectiveness chapter clear [Strongly disagree]

Or The following statements are about other sections of an ICER report that talk about uncertainty.... = I find the “Uncertainty and Controversies” sections of the cost-effectiveness chapter clear [Somewhat disagree]

Or The following statements are about other sections of an ICER report that talk about uncertainty.... = I find the “Uncertainty and Controversies” sections of the cost-effectiveness chapter clear [Neither agree nor disagree]

Q6.2 What are ways to make the "uncertainty and controversies" section in the cost-effectiveness chapter clearer?

____

____

____

____

____

Display this question:

If The following statements are about other sections of an ICER report that talk about uncertainty.... = I find the "Uncertainty and Controversies" section in the comparative clinical effectiveness chapter clear [Strongly disagree]

Or The following statements are about other sections of an ICER report that talk about uncertainty.... = I find the "Uncertainty and Controversies" section in the comparative clinical effectiveness chapter clear [Somewhat disagree]

Or The following statements are about other sections of an ICER report that talk about uncertainty.... = I find the "Uncertainty and Controversies" section in the comparative clinical effectiveness chapter clear [Neither agree nor disagree]

Q6.3 What are ways to make the "uncertainty and controversies" section in the comparative clinical effectiveness chapter clearer?

____

____

____

____

____

Display this question:

If The following statements are about other sections of an ICER report that talk about uncertainty.... = I find the cost-effectiveness summary and comment clear [Strongly disagree]

Or The following statements are about other sections of an ICER report that talk about uncertainty.... = I find the cost-effectiveness summary and comment clear [Somewhat disagree]

Or The following statements are about other sections of an ICER report that talk about uncertainty.... = I find the cost-effectiveness summary and comment clear [Neither agree nor disagree]

Q6.4 What are ways to make the "summary and comment" section clearer?

____

____

____

____

____

Display this question:

If The following statements are about other sections of an ICER report that talk about uncertainty.... = I find the health benefit price benchmark section clear [Strongly disagree]

Or The following statements are about other sections of an ICER report that talk about uncertainty.... = I find the health benefit price benchmark section clear [Neither agree nor disagree]

Or The following statements are about other sections of an ICER report that talk about uncertainty.... = I find the health benefit price benchmark section clear [Somewhat disagree]

Or The following statements are about other sections of an ICER report that talk about uncertainty.... = The impact of uncertainty on the health benefit price benchmark is clear [Strongly disagree]

Or The following statements are about other sections of an ICER report that talk about uncertainty.... = The impact of uncertainty on the health benefit price benchmark is clear [Somewhat disagree]

Or The following statements are about other sections of an ICER report that talk about uncertainty.... = The impact of uncertainty on the health benefit price benchmark is clear [Neither agree nor disagree]

Q30 What are ways to make the health benefit price benchmark section clearer?

____

____

____

____

____

Display this question:

If What research outputs from ICER do you read? Check all that apply. = ICER Evidence Reports (i.e., Draft, Revised, or Final)

Q6.6 Is there anything else you have not already mentioned about how ICER communicates uncertainty in their evidence reports that you would like to add?

____

End of Block: Topic 5: Understanding different sections of the ICER report

---

Start of Block: Topic 6: Understanding communication outside of ICER reports

Q7.1 The following statement is about ICER communications outside of their evidence reports. Examples of this are the patient snapshot or the report at a glance. If you do not read these communications, please select "Not applicable".

	Strongly disagree (1)	Somewhat disagree (2)	Neither agree nor disagree (3)	Somewhat agree (4)	Strongly agree (5)	Not applicable (6)
ICER communicates clearly about the results and uncertainty of the cost-effectiveness analysis outside of their evidence reports (1)	○	○	○	○	○	○

Q7.5 Do you think the communication about the results and uncertainty of the cost-effectiveness analysis outside of the ICER evidence reports can be clearer? If so, what would be ways to make this communication more clear?

____

End of Block: Topic 6: Understanding communication outside of ICER reports
